# Adjuvant therapeutic efficacy of low-dose aspirin on short-term outcomes of patients with cancer-associated venous thromboembolism

**DOI:** 10.1186/s12916-025-04284-8

**Published:** 2025-07-28

**Authors:** Wei Xiong, Zhenzhong Deng, Yi Cheng, Xiaoyang Song, Qihuan Yao, Jianmin Qu, Mei Xu, Fengfeng Han, Xuejun Guo, Yong Luo

**Affiliations:** 1 Department of Pulmonary and Critical Care Medicine, Xinhua Hospital, Shanghai Jiaotong University School of Medicine, Shanghai, China; 2 Department of Cardiovascular Medicine, Graduate School of Medicine, Kyoto University, Kyoto, Japan; 3 Department of Pulmonary and Critical Care Medicine, Chongming Hospital, Shanghai University of Medicine and Health Sciences, Shanghai, China; 4 Department of Oncology, Xinhua Hospital, Shanghai Jiaotong University School of Medicine, Shanghai, China; 5 Department of Traditional Chinese Medicine, Kongjiang Hospital, Yangpu District, Shanghai, China; 6 Department of Critical Care Medicine, Tongxiang First People’s Hospital, Zhejiang Province, Tongxiang, China; 7 Department of General Practice, Bund Community Health Service Center, Hongkou District, Shanghai, North China

**Keywords:** Venous thromboembolism, Cancer, Outcomes, Aspirin, Adjuvant

## Abstract

**Background:**

Although aspirin was reported to have primary thromboprophylactic efficacy on cancer patients, its adjuvant role in the treatment of patients with cancer-associated venous thromboembolism (VTE) has been unclear yet.

**Methods:**

Patients with cancer-associated VTE were retrospectively analyzed and divided into aspirin group and non-aspirin group based on whether they underwent low-dose aspirin (100 mg daily) in addition to conventional anticoagulants. Propensity score matching was used to balance baseline characteristics between the aspirin group and non-aspirin group in a 1:2 ratio. The primary, secondary, and tertiary outcomes were VTE recurrence, mortality and major bleeding, and net clinical benefit (NCB), at 6 months after VTE diagnosis, respectively.

**Results:**

The VTE recurrence occurred in 13 (3.1%) in the aspirin group (*N* = 423) and 55 (6.5%) in the non-aspirin group (*N* = 846) (hazard ratio [HR] 0.546, 95% confidence interval [CI] [0.298–0.988], *P* = 0.011). The PE-related mortality occurred in 12 (2.8%) in the aspirin group and 46 (5.4%) in the non-aspirin group (HR 0.535, 95% CI [0.283–0.909], *P* = 0.037). The all-cause mortality occurred in 108 (25.5%) in the aspirin group and 228 (27.0%) in the non-aspirin group (HR 0.983, 95% CI [0.782–1.237], *P* = 0.887). The major bleeding occurred in 52 (12.3%) in the aspirin group and 46 (5.4%) in the non-aspirin group (HR 2.448, 95% CI [1.646–3.641], *P* < 0.001). The NCB occurred in 274 (64.8%) in the aspirin group and 554 (65.5%) in the non-aspirin group (HR 0.976, 95% CI [0.801–1.189], *P* = 0.812).

**Conclusions:**

For patients with cancer-associated VTE, the adjuvant use of low-dose aspirin based on conventional anticoagulants improves VTE recurrence and PE-related mortality, compared with isolated use of anticoagulants, whereas it does not improve all-cause mortality or net clinical benefit. Adjuvant low-dose aspirin use is associated with an increased risk of bleeding.

**Supplementary Information:**

The online version contains supplementary material available at 10.1186/s12916-025-04284-8.

## Background

As a result of the growing and aging population and increases in cancer survival due to advances in early detection and treatment, the impact of cancer to human health is still ongoing. [1] For active cancer patients, cancer-associated venous thromboembolism (VTE) that comprises pulmonary embolism (PE) and deep vein thrombosis (DVT) is their second leading cause of death after the cancer per se. [2, 3]

Aspirin, also known as acetylsalicylic acid, is an antithrombotic agent due to its inhibitory activity of platelet aggregation. Low-dose aspirin that is defined as 100 mg daily is endorsed for the thromboprophylaxis of cancer-associated VTE in multiple myeloma by several mainstream guidelines. [3–6] Furthermore, it was reported that aspirin was also associated with lower VTE incidence without significant bleeding risk, for patients with lung, stomach, colon, liver, pancreatic, prostate, breast, ovarian, bladder, and kidney cancers, as well as lymphoma and leukemia. [7, 8] Aspirin is considered to have a primary thromboprophylactic efficacy on VTE occurrence in patients with active cancer. In addition, it was reported that use of aspirin can safely reduce cancer incidence, cancer mortality, and cancer metastatic risk. [9–12] Aspirin is also believed to have anticancer activity.

Nevertheless, the secondary thromboprophylactic efficacy of aspirin on VTE recurrence in patients diagnosed with cancer-associated VTE is still unclear currently, since previous studies have suggested that aspirin has moderate efficacy in the secondary thromboprophylaxis of VTE recurrence in general VTE patients. [13, 14] Given that aspirin has primary thromboprophylaxis and anticancer efficacy on cancer patients, it was hypothesized that the joint use of low-dose aspirin on the basis of conventional anticoagulation therapy may have certain adjuvant therapeutic efficacy, thereby improving the overall outcomes of patients with cancer-associated VTE, compared with isolated conventional anticoagulation therapy. Due to the current scarcity of such literature, we conducted the current study to address this topic.

## Methods

### Study design

The current investigation was an investigator-initiated, multicenter, retrospective, observational cohort study that investigated the adjuvant therapeutic efficacy of low-dose aspirin on the clinical outcomes of patients with acute cancer-associated VTE. International Classification of Diseases, 10th revision (ICD-10) codes were used to identify subjects. We selected consecutive patients with the ICD-10 codes of I26 (I26.0, I26.9) or I80 (I80.1, I80.2, I80.3) or I82 (I82.2, I82.3, I82.4, I82.6, I82.8, I82.9), and with the ICD-10 codes among C00–C97 concurrently, from the inpatient electronic medical record systems of the participating medical centers. Cancer-associated VTE patients who underwent conventional anticoagulation treatment with and without low-dose oral aspirin regimen for 6 months after the diagnosis of cancer-associated VTE were collected. The patients who took low-dose aspirin daily were defined as aspirin group, whereas those who did not take aspirin or took aspirin occasionally were defined as non-aspirin group. Low-dose aspirin was defined as 100 mg daily. [3–6] According to the presence or absence of PE, all patients were further divided into PE and non-PE subgroups. The aspirin prescription was fully at the discretion of the attending physicians of all patients, for the purpose of primary or secondary prophylaxis of cardiovascular and/or cerebrovascular events in most cases.

### Patients

The inclusion criteria were as follows: (1) Patients were 18 years old or older. (2) Patients had primary active cancer [15] with definite pathological diagnosis and objectively confirmed diagnosis of acute cancer-associated VTE, by means of compression ultrasonography (CUS) on lower extremities, computed tomography pulmonary angiography (CTPA), and planar ventilation/perfusion (V/Q) scan. [16] (3) Patients underwent a standard treatment of conventional direct oral anticoagulants (DOACs) which included rivaroxaban, edoxaban, and dabigatran for at least 6 months after the diagnosis of cancer-associated VTE, [16–18] with or without taking low-dose aspirin, and underwent routine VTE follow-up once every 3 to 6 months in principle according to the guidelines, [2–6, 19] at the discretion of their attending physicians. (4) Patients received appropriate anticancer treatment in line with the recommendation from the Chinese Society of Clinical Oncology (CSCO), at the discretion of their attending oncologists. The exclusion criteria were as follows: (1) Patient had a definite medical history of chronic thromboembolic disease (CTED). [16] (2) Patients received non-low-dose aspirin or other antiplatelet agents than aspirin during the treatment of cancer-associated VTE.

### Follow-up

The patients entered follow-up stage immediately after discharge. They were requested to return to the same hospitals where their VTE was diagnosed and treated for follow-up visits at 3 and 6 months after cancer-associated VTE diagnosis. All the patients were required to undergo mandatory VTE imaging review at the 3-month follow-up after cancer-associated VTE diagnosis. The patients also needed to undergo VTE imaging review if they presented with typical VTE recurrent symptoms, between the 3-month and 6-month follow-up visits after cancer-associated VTE diagnosis. All information of the patients were retrieved from the electronic medical record system of the participating hospitals. Patients whose crucial information were lacking in the electronic medical record system were contacted by telephone to supplement relevant information.

### Outcome measures

The diagnosis of cancer-associated VTE was defined as baseline. The primary outcome of the current study was VTE recurrence including PE, DVT, or both, at 6 months after the cancer-associated VTE diagnosis. VTE recurrence was defined as new defect on V/Q scan or new thromboembolism on CTPA, or new thrombi in venous segments or an increase more than 4 mm in venous diameter for a previous thrombi on CUS, by comparison with previous imaging, [20] with or without the symptoms of recurrence. The secondary outcomes were mortality including PE-related and all-cause mortality, and major bleeding, at 6 months after the cancer-associated VTE diagnosis. PE-related mortality was defined as objectively confirmed PE before death without more likely cause of death, autopsy-confirmed PE without more likely cause of death, or PE was most likely the major cause of death albeit being not objectively confirmed. [21] Major bleeding was defined as fatal bleeding, symptomatic bleeding in a critical area or organ, or bleeding leading to a fall of 20 g/L or more in hemoglobin level, or to a transfusion of two or more units of whole blood or red cells. [22] The tertiary outcome was net clinical benefit (NCB) that was defined as the patients without any one of the VTE recurrence, major bleeding, or death from any cause, [13] at 6 months after the cancer-associated VTE diagnosis.

### Study oversight

The current study protocol was approved by the institutional review board of Shanghai Xinhua Hospital (approval number: XHEC-QT-2021–056). Shanghai Xinhua Hospital, Shanghai Chongming District Central Hospital, Shanghai Kongjiang Hospital, Tongxiang First People’s Hospital, and Shanghai North Bund Community Health Service Center participated in the current study. Written informed consent from the participants or their next of kin was waived due to: (1) the study involves no more than minimal risk for patients; (2) the study cannot adversely affect the rights and welfare of patients; (3) the study cannot be performed without the exemption of informed consent of patients. All the authors contributed to the final manuscript and guaranteed the accuracy and completeness of the data and the fidelity of the study to the protocol. No one who is not an author contributes to the manuscript. The overall reporting of this study was basically in line with the Strengthening the Reporting of Observational Studies in Epidemiology (STROBE) statement. [23]

### Statistical analysis

Continuous variables were presented as mean and standard deviation or as median and interquartile range, based on whether they followed a normal distribution, whereas categorical variables were presented as numbers and percentages. Shapiro–Wilk test was used in the normality test. Missing data were addressed using multiple imputation. Comparison of continuous variables were performed using Student *t*-test, or Mann–Whitney *U* test based on whether they followed a normal distribution, whereas comparison of categorical variables were conducted using χ2 test or Fisher exact test. Time-dependent cumulative incidences of outcomes between the aspirin and non-aspirin groups for all the patients and for the PE subgroup were compared using Kaplan–Meier method. Fine and Gray regression model [24] was used to compute the hazard ratio (HR) and two-sided 95% confidence intervals (CI) for the outcome comparison between the aspirin and non-aspirin groups after adjustment for the competing risk of death unrelated to VTE.

Propensity score matching (PSM) was used to eliminate the potential bias in the current study. Basically based on the previous literature, [16] we matched the demographics including age, sex, and body mass index, VTE recurrence-related factors including Ottawa score, previous VTE, thrombophilia, PE occurrence, cancer types mainly including lung, lymphoma, gynecologic, bladder, brain, stomach, and pancreatic cancers, [2] D-dimer, thromboprophylaxis before VTE, anticancer treatment after VTE, and types of DOACs used after VTE, mortality-related factors including high-risk PE, cardiopulmonary diseases, terminal cancer, metastasis, and performance status, and bleeding-related factors including major bleeding history and VTE-BLEED score, between the aspirin and non-aspirin groups. Nearest-neighbor matching, also known as greedy matching, was used without replacement in the PSM analysis. [25] The ratio of number of patients in the aspirin group to non-aspirin group was predetermined to be 1:2, since the number of patients in the non-aspirin group was much more than that in the aspirin group. We selected 1 patient in the aspirin group first, then matched 2 patients in the non-aspirin group who had a linear propensity score being closest to the selected one in the aspirin group, using calipers of width equal to 0.2 of the standard deviation of the logit of the propensity score. [26] SPSS 26 and R software 3.6.1 were used for statistical analysis. Statistical significance was defined as a *P* value being less than 0.05.

## Results

### Patients

**Fig. 1:**
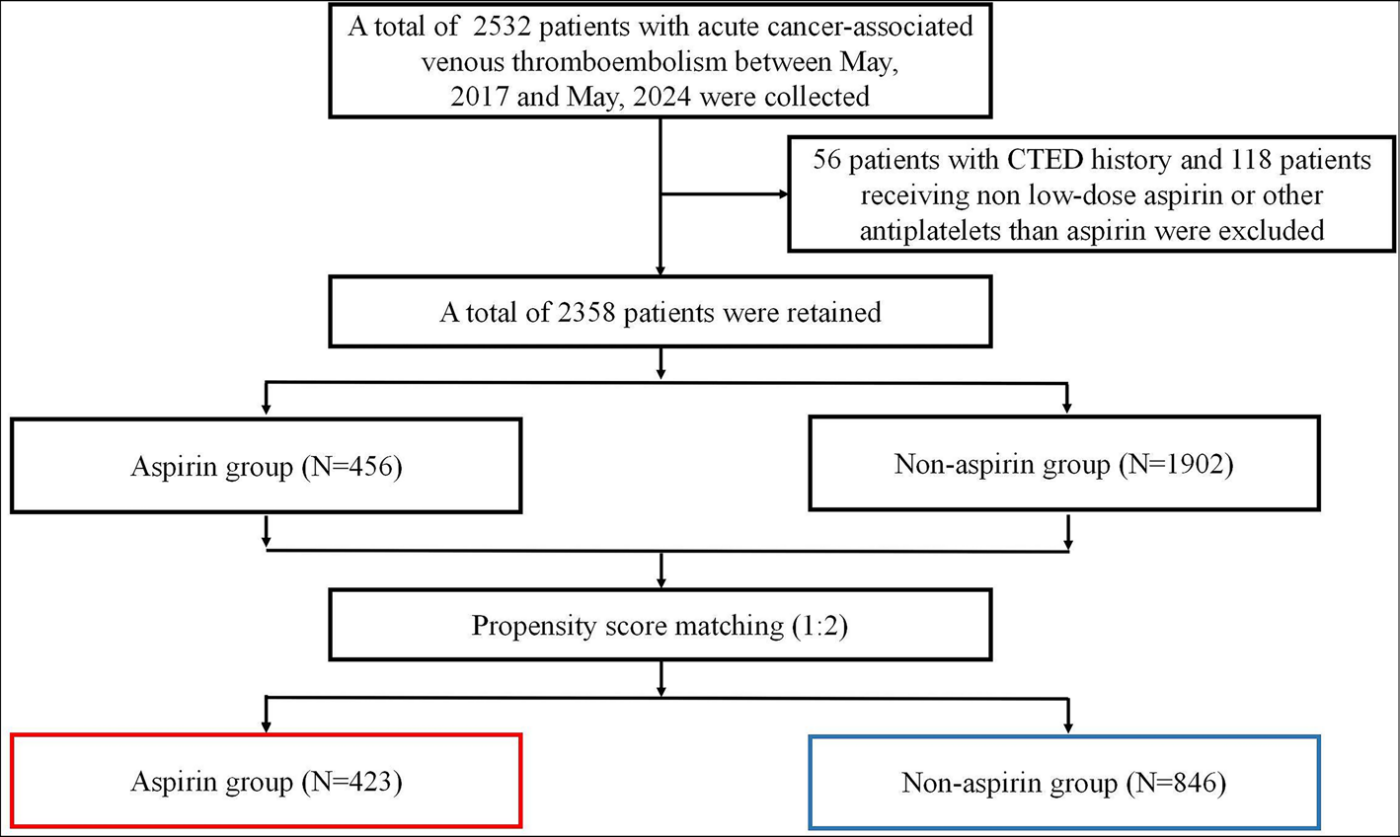
Study flowchart. Abbreviation: CTED, chronic thromboembolic disease

A total of 2532 patients between May 2017 and May 2024 in participating centers were collected according to the inclusion criteria. After excluding 56 patients with CTED history and 118 patients receiving non-low-dose aspirin or other antiplatelets than aspirin according to the exclusion criteria, there were 2358 patients left to proceed to the next research step. After PSM, there were 1269 patients who have entered the final analysis stage. The number of patients in the aspirin group and non-aspirin group were 423 and 846, respectively. The study flowchart is demonstrated in Fig. 1. The Love plot demonstrating absolute standardized mean difference between the aspirin and non-aspirin groups before and after PSM is displayed in Fig. S1 in Additional file 1.

There were no significantly statistical difference in demographics, VTE recurrence-related factors, mortality-related factors, and bleeding-related factors, between the two groups at baseline. The characteristics of patients after PSM are presented in Table 1. The characteristics of patients before PSM are presented in Table S1 in Additional file 2. Cancer types in detail after PSM are presented in Table S2 in Additional file 2.

**Table 1:** Characteristics of patients after propensity score matching

	**Aspirin (*N* = 423)**	**Non-aspirin (*N* = 846)**	***P* value**
**Demographics**			
Age—years	67.8 ± 11.5	68.9 ± 11.4	0.108
Male—no. (%)	252 (59.6)	543 (64.2)	0.110
Body mass index—kg/m^2^	22.2 ± 3.7	22.4 ± 4.1	0.335
**Medical history—no. (%)**			
VTE history	23 (5.4)	38 (4.5)	0.458
Cardiovascular diseases	194 (45.9)	40 (4.7)	< 0.001
Cerebrovascular diseases	137 (32.4)	52 (6.1)	< 0.001
Thrombophilia	17 (4.0)	26 (3.1)	0.380
Active autoimmune diseases	19 (4.5)	37 (4.4)	0.923
Chronic pulmonary diseases	39 (9.2)	83 (9.8)	0.736
Major bleeding history	57 (13.5)	104 (12.3)	0.551
**VTE characteristics**			
PE without DVT—no. (%)	89 (21.0)	180 (21.3)	0.923
PE with DVT—no. (%)	81 (19.1)	201 (23.8)	0.063
Isolated DVT—no. (%)	253 (59.8)	465 (55.0)	0.101
Symptomatic VTE—no. (%)	222 (52.5)	475 (56.1)	0.216
High-risk PE—no. (%)	12 (2.8)	28 (3.3)	0.650
Ottawa score—points	0.27 ± 0.80	0.20 ± 0.86	0.141
VTE-BLEED score—points	2 (1–3)	2 (1–3)	0.396
**Cancer characteristics**			
Metastatic cancer—no. (%)	103 (24.3)	240 (28.4)	0.129
Terminal cancer—no. (%)	32 (7.6)	85 (10.0)	0.150
Recurrent cancer—no. (%)	51 (12.1)	89 (10.5)	0.410
Performance status—points	0 (0–1)	0 (0–1)	0.199
Anticancer treatment after VTE—no. (%)	306 (72.3)	582 (68.8)	0.194
Surgery	112 (26.5)	253 (29.9)	0.203
Chemotherapy	246 (58.2)	451 (53.3)	0.102
Tyrosine kinase inhibitors	137 (32.4)	244 (28.8)	0.194
Immunotherapy	96 (22.7)	158 (18.7)	0.092
**Laboratory tests**			
D-dimer—mg/L	3.2 ± 2.1	3.3 ± 2.3	0.357
Platelet—10^9^/L	212 ± 96	218 ± 92	0.306
Hemoglobin—g/L	110 ± 18	112 ± 19	0.092
Creatine—μmol/L	75.8 ± 59.7	72.0 ± 36.6	0.230
**Anticoagulants—no. (%)**			
Dabigatran	66 (15.6)	144 (17.0)	0.642
Edoxaban	136 (32.1)	289 (34.2)	0.475
Rivaroxaban	221 (52.2)	413 (48.8)	0.250
Thromboprophylaxis before VTE	105 (24.8)	221 (26.1)	0.617
**Aspirin—no. (%)**			
Long term—no. (%)	327 (77.3)	52 (6.1)	< 0.001
Length of use—years	16.8 ± 8.2	3.6 ± 2.7	< 0.001

Continuous variables are presented as “mean ± standard deviation” or as “median (interquartile range),” based on whether they follow a normal distribution

All other variables were overall complete, except BMI and VTE-BLEED score were processed by multiple imputation

Anticancer treatment was defined as a composite of medical and surgical therapies against cancer

Due to the fact that the same patient may receive multiple anticancer therapies, the sum of the proportions of several anticancer regimens in this table may exceed the proportion of the total anticancer treatment

Cancer types are presented in Table S2 in the Supplementary Material

Long-term aspirin use was defined as patients being prescribed with aspirin for at least 6 months

Abbreviations: *VTE*, venous thromboembolism; *PE*, pulmonary embolism; *DVT*, deep vein thrombosis; *VTE-BLEED*, actiVe cancer, male with uncontrolled hyperTension at baseline, anaEmia, history of BLeeding, agE ≥ 60 years, rEnal Dysfunction

### Outcomes

**Fig. 2:**
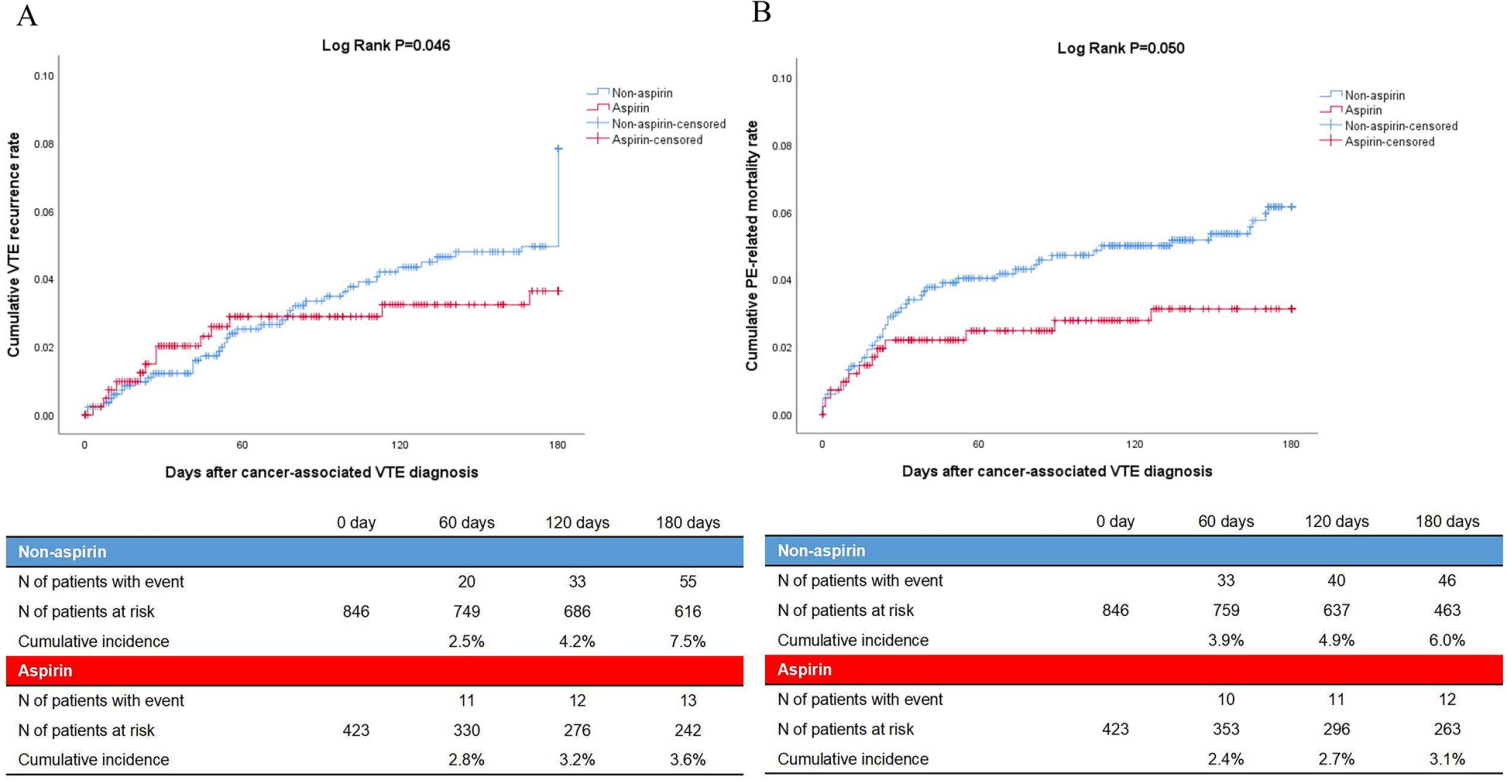
Comparison of time-dependent VTE recurrence and PE-related mortality between aspirin and non-aspirin groups. Abbreviations: VTE, venous thromboembolism; PE, pulmonary embolism

The primary outcome which was VTE recurrence occurred in 13 of 423 patients (3.1%) in the aspirin group and in 55 of 846 patients (6.5%) in the non-aspirin group (HR 0.546, 95% CI [0.298–0.988], *P* = 0.011) (Table 2). The recurrent isolated PE, PE with DVT, and isolated DVT were 4 (30.8%), 2 (15.4%), and 7 (53.8%) in the aspirin group and 13 (23.6%), 16 (29.1%), and 26 (47.3%) in the non-aspirin group, respectively (*P* = 0.590). Pairwise comparison demonstrated that there was no statistical difference in the proportion of each type of VTE recurrence between the two groups either. The time-dependent occurrence of VTE recurrence is shown in Fig. 2A (log rank *P* = 0.046).

**Table 2:** Comparison of outcomes between aspirin and non-aspirin groups

	**Aspirin (*N* = 423)**	**Non-aspirin (*N* = 846)**	**Hazard ratio (95% confidence interval)**	***P* value**
**Primary outcome—no. (%)**				
VTE recurrence	13 (3.1)	55 (6.5)	0.546 (0.298–0.988)	0.011
**Secondary outcomes—no. (%)**				
PE-related mortality	12 (2.8)	46 (5.4)	0.535 (0.283–0.909)	0.037
All-cause mortality	108 (25.5)	228 (27.0)	0.983 (0.782–1.237)	0.887
Major bleeding	52 (12.3)	46 (5.4)	2.448 (1.646–3.641)	< 0.001
**Tertiary outcome—no. (%)**				
Net clinical benefit	274 (64.8)	554 (65.5)	0.976 (0.801–1.189)	0.812

Net clinical benefit was defined as the patients without any one of the VTE recurrence, major bleeding, or death from any cause

Due to the possibility of VTE recurrence, major bleeding, or all-cause death occurring simultaneously in the same patient, the sum of the proportions of VTE recurrence, all-cause death, major bleeding, and net clinical benefit may exceed 100%

Abbreviations: *VTE*, venous thromboembolism; *PE*, pulmonary embolism

The PE-related mortality in the secondary outcomes occurred in 12 of 423 patients (2.8%) in the aspirin group and in 46 of 846 patients (5.4%) in the non-aspirin group (HR 0.535, 95% CI [0.283–0.909], *P* = 0.037) (Table 2). The time-dependent occurrence of PE-related mortality events is also shown in Fig. 2B (log rank *P* = 0.050). The all-cause mortality in the secondary outcomes occurred in 108 of 423 patients (25.5%) in the aspirin group and in 228 of 846 patients (27.0%) in the non-aspirin group (HR 0.983, 95% CI [0.782–1.237], *P* = 0.887) (Table 2). The time-dependent occurrence of all-cause mortality events is shown in Fig. S2 in Additional file 1 (log rank *P* = 0.886). The major bleeding in the secondary outcomes occurred in 52 of 423 patients (12.3%) in the aspirin group and in 46 of 846 patients (5.4%) in the non-aspirin group (HR 2.448, 95% CI [1.646–3.641], *P* < 0.001) (Table 2). The time-dependent occurrence of major bleeding events is shown in Fig. S3 in Additional file 1 (log rank *P* < 0.001).

The tertiary outcome which was NCB occurred in 274 of 423 patients (64.8%) in the aspirin group and in 554 of 846 patients (65.5%) in the non-aspirin group (HR 0.976, 95% CI [0.801–1.189], *P* = 0.812) (Table 2).

### PE subgroup

For the PE subgroup which included 749 patients with cancer-associated PE, the primary outcome occurred in 9 of 235 patients (3.8%) in the aspirin group and in 33 of 514 patients (6.4%) in the non-aspirin group (HR 0.682, 95% CI [0.326–1.426], *P* = 0.309) (Table 3). The PE-related mortality in the secondary outcomes occurred in 8 of 235 patients (3.4%) in the aspirin group and in 35 of 514 patients (6.8%) in the non-aspirin group (HR 0.504, 95% CI [0.234–1.087], *P* = 0.081) (Table 3). The all-cause mortality in the secondary outcomes occurred in 60 of 235 patients (25.5%) in the aspirin group and in 144 of 514 patients (28.0%) in the non-aspirin group (HR 0.930, 95% CI [0.688–1.256], *P* = 0.635) (Table 3). The major bleeding in the secondary outcomes occurred in 28 of 235 patients (11.9%) in the aspirin group and in 28 of 514 patients (5.4%) in the non-aspirin group (HR 2.316, 95% CI [1.371–3.911], *P* = 0.002) (Table 3). The NCB occurred in 153 of 235 patients (65.1%) in the aspirin group and in 332 of 514 patients (64.6%) in the non-aspirin group (HR 1.032, 95% CI [0.795–1.340], *P* = 0.813) (Table 3).

**Table 3:** Comparison of outcomes between aspirin and non-aspirin groups in PE subgroup

	**Aspirin (*N* = 235)**	**Non-aspirin (*N* = 514)**	**Hazard ratio (95% confidence interval)**	***P* value**
**Primary outcome—no. (%)**				
VTE recurrence	9 (3.8)	33 (6.4)	0.682 (0.326–1.426)	0.309
**Secondary outcomes—no. (%)**				
PE-related mortality	8 (3.4)	35 (6.8)	0.504 (0.234–1.087)	0.081
All-cause mortality	60 (25.5)	144 (28.0)	0.930 (0.688–1.256)	0.635
Major bleeding	28 (11.9)	28 (5.4)	2.316 (1.371–3.911)	0.002
**Tertiary outcome—no. (%)**				
Net clinical benefit	153 (65.1)	332 (64.6)	1.032 (0.795–1.340)	0.813

Net clinical benefit was defined as the patients without any one of the VTE recurrence, major bleeding, or death from any cause

Due to the possibility of VTE recurrence, major bleeding, or all-cause death occurring simultaneously in the same patient, the sum of the proportions of VTE recurrence, all-cause death, major bleeding, and net clinical benefit may exceed 100%

Abbreviations: *VTE*, venous thromboembolism; *PE*, pulmonary embolism

## Discussion

Major findings derived from the current results were as follows: (1) For patients with cancer-associated VTE, the joint use of low-dose aspirin and anticoagulants reduced 6-month VTE recurrence and PE-related mortality rates, compared with isolated use of anticoagulants. (2) The joint use of low-dose aspirin and anticoagulants did not improve the 6-month all-cause mortality rate or NCB, compared with isolated use of anticoagulants. (3) The joint use of low-dose aspirin and anticoagulants raised 6-month major bleeding rate, compared with isolated use of anticoagulants. To our best knowledge, this is the first study conducted on this topic, since there have been no comparable ones so far.

Although the primary thromboprophylactic efficacy of aspirin on cancer patients had been extensively reported, [7, 8, 27] its role in the treatment of cancer-associated VTE remained unclear before the current study. In the ASPIRE study, [13] the use of aspirin did not significantly reduce the rate of isolated VTE recurrence, in patients with unprovoked VTE who had completed initial anticoagulant therapy. Nevertheless, since cancer patients accounted for merely 1.2% of all patients in the ASPIRE study, [13] the findings cannot be fully extrapolated to patients with cancer-associated VTE. The current study suggested that aspirin had certain short-term adjuvant therapeutic efficacy on the secondary prophylaxis of VTE recurrence and the improvement of PE-related mortality in cancer-associated VTE patients, when it was used in combination with anticoagulant agents. This may be attributed to two reasons. One is that aspirin per se could have certain antithrombotic efficacy for the secondary prophylaxis of VTE recurrence. [13, 14] The other is that the joint use of aspirin and anticoagulant agents may produce a synergistic effect, leading to a result greater than the simple sum of their therapeutic efficacy. [28]

One noteworthy point is that most patients in the aspirin group had a long-term history of aspirin use which was longer than 6 months, [29] compared with the patients in the non-aspirin group, which was natural in a retrospective study. This may raise three issues. One is that since the patients in the aspirin group still experienced VTE development despite undergoing primary thromboprophylaxis with aspirin, it suggested that these patients might have a higher risk of thrombosis due to comorbid cardiovascular and/or cerebrovascular diseases, compared with those in the non-aspirin group. The other issue is whether the aspirin use of the patients in the aspirin group before VTE diagnosis affect their outcomes after VTE diagnosis. In our opinion, since the use of aspirin had already failed to prevent VTE from occurring for the patients in the aspirin group, the aspirin use before VTE diagnosis may have little impact on their outcomes after VTE diagnosis. The last issue is that since the use of aspirin had failed in the primary thromboprophylaxis for the patients in the aspirin group, why did it succeed later in the secondary thromboprophylaxis for these patients? In our opinion, we deem that the synergistic effect produced by the joint use of aspirin and anticoagulant agents [28] may be the reason for this phenomenon.

Although many studies have reported that taking aspirin has anticancer efficacy and can reduce cancer mortality, [9–12] the use of adjuvant aspirin did not improve the 6-month all-cause mortality of cancer patients in this study. Our finding is consistent with that in the study of Chen et al., in which daily aspirin therapy did not improve the risk of cancer recurrence or survival in early follow-up of patients with high-risk nonmetastatic breast cancer. [30] Of note, the average follow-up time ranged from 4 to 12 years in the previous studies in which aspirin had anticancer efficacy, [9–12] whereas were merely 34 months and 6 months in the study of Chen et al. [30] and in the current study, respectively. This may be the reason why aspirin did not improve the all-cause mortality in this study and in the study of Chen et al. [30]

There was no significant difference in 6-month NCB comprising VTE recurrence, major bleeding, and all-cause mortality between the aspirin and non-aspirin groups in this study. Although the aspirin group had lower VTE recurrence rate, it had higher major bleeding rate and similar all-cause mortality rate, compared with the non-aspirin group. Accordingly, it is not surprising that there was no significant difference in NCB between the two groups. In addition, being inconsistent with the main findings, aspirin did not improve VTE recurrence or PE-related mortality rates for the patients in the PE subgroup. Of note, unlike the overall patient population, the baseline characteristics did not match very well between the aspirin and non-aspirin groups in the PE subgroup. So this result needs to be interpreted with caution.

The clinical implication of the current findings needs to be interpreted carefully. In terms of reducing short-term VTE recurrence and PE-related mortality rates alone, it is recommended to use aspirin on the basis of conventional anticoagulants in patients with cancer-associated VTE. However, in terms of reducing short-term all-cause mortality, major bleeding incidence, and improving NCB, the use of aspirin on the basis of conventional anticoagulants does not benefit, or even harm patients with cancer-associated VTE. In summary, caution should be exercised when using aspirin as an adjuvant therapy for improving the short-term outcomes of cancer-associated VTE patients, weighing pros and cons, in order to maximize the benefits to patients.

### Limitations

There are some limitations that must be acknowledged in this study. First, although PSM was used in this study, the retrospective assessment of drug efficacy is subject to inherent biases such as potential between-group imbalance that prevents definitive conclusions. Instead, the current study should be considered as a hypothesis-generating pilot study for the randomized controlled trials (RCTs) warranted in the future. Second, due to the relatively small sample size of this study, if the patients are divided into subgroups based on different cancer types representing different VTE risks, the sample size in each subgroup would be inconsiderable. Therefore, subgroup analysis based on cancer types was not conducted in the current study. Third, the follow-up procedures were not standardized across centers, which is common in retrospective studies even in some multicenter prospective studies. Nevertheless, the basic principles for the follow-up of patients with cancer-associated VTE in the medical centers were all in line with the guidelines. [2–6, 19] Fourth, since most of the patients in the aspirin group had comorbidities of cardiovascular and cerebrovascular diseases, while those in the non-aspirin group did not, it is impracticable to eliminate this bias with PSM, otherwise the sample size would be greatly shrunk. It would be best to eliminate this confounding factor in the future RCTs. However, apart from cardiovascular and cerebrovascular comorbidities, other confounding factors were well matched between the two groups. Fifth, the long-term aspirin use among the patients in the aspirin group may have certain impact on the results. It may be necessary to select patients without a history of aspirin use for the validation of current findings in the future RCTs. Sixth, the follow-up period of this study was 6 months, since the initial treatment course for cancer-associated VTE is usually 6 months. [3–5, 16] In this context, the impact of aspirin on the long-term outcomes of cancer-associated VTE patients 6 months after VTE diagnosis is currently unknown. Seventh, although the definition of NCB in the current study is conventional, it could be flawed in cancer patients who have relatively high mortality rate. Incorporating all-cause death into the NCB does not allow for the assessment of potential competing risks between fatal and non-fatal events. However, NCB was merely the tertiary outcome in the current study. Last, due to the high usage rate of DOACs and low usage rate of low molecular weight heparin (LMWH) and vitamin K antagonist (VKA) for the first 6-month treatment of cancer-associated VTE in China, all patients in this study received DOACs anticoagulant therapy. Therefore, caution should be exercised when extrapolating the current findings to cancer-associated VTE patients receiving LMWH and VKA.

## Conclusions

In conclusion, this study indicates for the first time that the adjuvant use of low-dose aspirin based on conventional anticoagulants reduces short-term VTE recurrence and PE-related mortality rates in patients with cancer-associated VTE, compared with using isolated anticoagulants. However, the joint use of low-dose aspirin and conventional anticoagulants cannot improve short-term all-cause mortality rate or net clinical benefit. Adjuvant low-dose aspirin use is associated with an increased risk of bleeding. The current findings may provide a new treatment method for adjuvant therapy of patients with cancer-associated VTE. Relevant RCTs could be warranted in the future.

## Supplementary Information

Additional file 1: Figs. S1–S3. Fig. S1 Love plot demonstrating absolute standardized mean difference between aspirin and non-aspirin groups before and after PSM. Fig. S2 Comparison of time-dependent all-cause mortality between aspirin and non-aspirin groups. Fig. S3 Comparison of time-dependent major bleeding between aspirin and non-aspirin groups. 

Additional file 2: Tables S1 and S2.

## Data Availability

Data are available upon reasonable request.

## Abbreviations

VTE: Venous thromboembolism
NCB: Net clinical benefit
HR: Hazard ratio
PE: Pulmonary embolism
DVT: Deep vein thrombosis
ICD-10: International Classification of Diseases, 10th revision
CUS: Compression ultrasonography
CTPA: Computed tomography pulmonary angiography
V/Q: Planar ventilation/perfusion
DOACs: Direct oral anticoagulants
CSCO: Chinese Society of Clinical Oncology
CTED: Chronic thromboembolic disease
STROBE: Strengthening the Reporting of Observational Studies in Epidemiology
CI: Confidence intervals
PSM: Propensity score matching
RCTs: Randomized controlled trials
LMWH: Low molecular weight heparin
VKA: Vitamin K antagonist
