# Supplementary material for: Adjuvant therapeutic efficacy of low-dose aspirin on short-term outcomes of patients with cancer-associated venous thromboembolism
Source: BMC Med. 2025 Jul 28;23:444. doi: 10.1186/s12916-025-04284-8 (PMC12306065; doi:10.1186/s12916-025-04284-8)
Supplement: Supplementary file 1 — Additional file 1: Figs. S1–S3. Fig. S1 Love plot demonstrating absolute standardized mean difference between aspirin and non-aspirin groups before and after PSM. Fig. S2 Comparison of time-dependent all-cause mortality between aspirin and non-aspirin groups. Fig. S3 Comparison of time-dependent major bleeding between aspirin and non-aspirin groups. [file 12916_2025_4284_MOESM1_ESM.docx]

**Additional Files**

**Additional File 1**

**
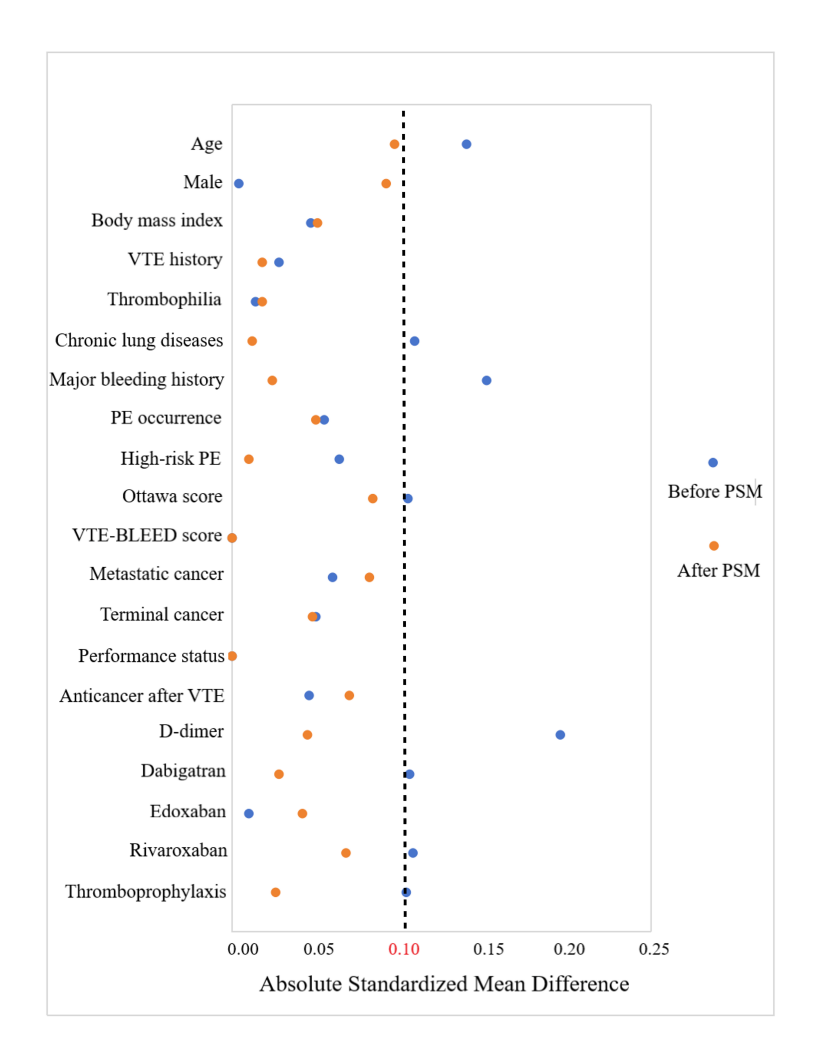
**

**Figure S1- Love plot demonstrating absolute standardized mean difference between aspirin and non-aspirin groups before and after PSM**

Abbreviation: PSM: propensity score matching; VTE: venous thromboembolism; PE: pulmonary embolism; VTE-BLEED: actiVe cancer, male with uncontrolled hyperTension at baseline, anaEmia, history of BLeeding, agE ≥ 60 years, rEnal Dysfunction


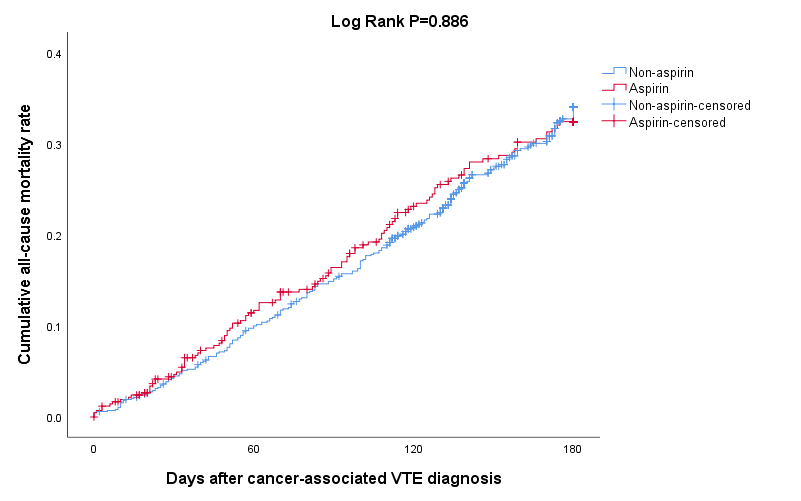


**Figure S2** **- Comparison of time-dependent all-cause mortality between aspirin and non-aspirin groups**

Abbreviation: VTE: venous thromboembolism


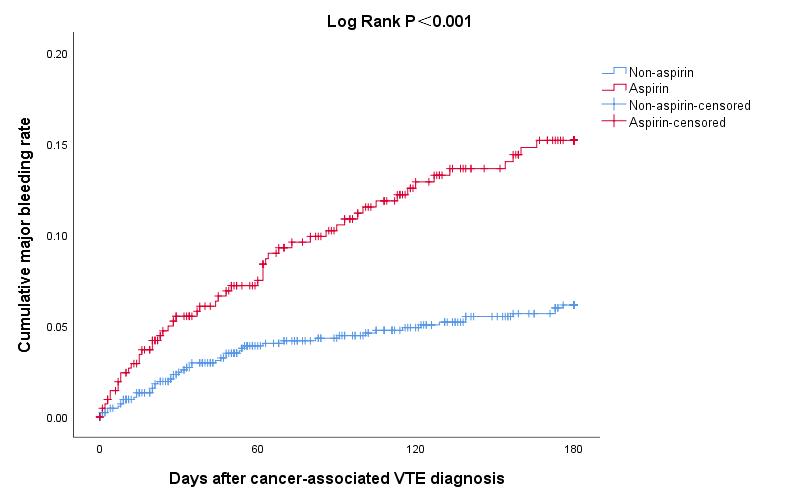


**Figure S3 - Comparison of time-dependent major bleeding between aspirin and non-aspirin groups**

Abbreviation: VTE: venous thromboembolism

**Additional File 2**

| **Table S1 -** **Characteristics of patients** **before propensity score matching** | | | |
| --- | --- | --- | --- |
|  | **Aspirin**  **(N=456)** | **Non-aspirin**  **(N=1902)** | **P value** |
| **Demographics** |  |  |  |
| Age - years | 69.0 ±14.3 | 66.9 ± 15.8 | 0.007 |
| Male - no. (%) | 278 (61.0) | 1156 (60.8) | 0.942 |
| Body mass index - kg/m^2^ | 23.1 ± 4.2 | 23.3 ± 4.4 | 0.297 |
| **Medical history** **- no. (%)** |  |  |  |
| VTE history | 31 (6.8) | 102 (5.4) | 0.233 |
| Cardiovascular diseases | 202 (44.3) | 48 (2.5) | <0.001 |
| Cerebrovascular diseases | 141 (30.9) | 63 (3.3) | <0.001 |
| Thrombophilia | 17 (3.7) | 84 (4.4) | 0.514 |
| Active autoimmune diseases | 34 (7.5) | 126 (6.6) | 0.526 |
| Chronic pulmonary diseases | 54 (11.8) | 138 (7.3) | 0.001 |
| Major bleeding history | 65 (14.3) | 128 (6.7) | <0.001 |
| **VTE characteristics** |  |  |  |
| PE without DVT - no. (%) | 118 (25.9) | 459 (24.1) | 0.436 |
| PE with DVT - no. (%) | 83 (18.2) | 485 (25.5) | 0.001 |
| Isolated DVT - no. (%) | 255 (55.9) | 958 (50.4) | 0.033 |
| Symptomatic VTE - no. (%) | 329 (72.1) | 1297 (68.2) | 0.101 |
| High-risk PE - no. (%) | 15 (3.3) | 124 (6.5) | 0.009 |
| Ottawa score - points | 0.28 ± 0.79 | 0.20 ± 0.83 | 0.048 |
| VTE-BLEED score - points | 2 (1-3) | 2 (1-3) | 0.196 |
| **Cancer characteristics** |  |  |  |
| Metastatic cancer | 110 (24.1) | 516 (27.1) | 0.192 |
| Terminal cancer | 35 (7.7) | 98 (5.2) | 0.036 |
| Recurrent cancer | 54 (11.8) | 216 (11.4) | 0.118 |
| Performance status | 0 (0-1) | 0 (0-1) | 0.056 |
| Anticancer treatment after VTE | 339 (74.3) | 1370 (72.0) | 0.321 |
| **Laboratory tests** |  |  |  |
| D-dimer - mg/L | 3.0 (2.9-6.0) | 3.9 (2.9-6.6) | 0.010 |
| Platelet - 10^9^/L | 205 ± 87 | 232 ± 92 | <0.001 |
| Hemoglobin - g/L | 118 ± 24 | 121 ± 24 | 0.055 |
| Creatine - μmol/L | 80.0 ± 54.4 | 76.1 ± 57.2 | 0.174 |
| **Anticoagulants** **- no. (%)** |  |  |  |
| Dabigatran | 91 (20.0) | 286 (15.0) | 0.010 |
| Edoxaban | 139 (30.5) | 570 (30.0) | 0.830 |
| Rivaroxaban | 226 (49.6) | 1046 (55.0) | 0.037 |
| Thromboprophylaxis before VTE | 136 (29.8) | 666 (35.0) | 0.036 |
| **Aspirin - no. (%)** |  |  |  |
| Long term - no. (%) | 341 (74.8) | 93 (4.9) | <0.001 |
| Length of use - years | 15.6 ± 7.7 | 3.3 ± 1.9 | <0.001 |

Continuous variables are presented as “mean ± standard deviation” or as “median (interquartile range)”, based on whether they follow a normal distribution.

All other variables were overall complete, except BMI and VTE-BLEED score were processed by multiple imputation.

Anticancer treatment was defined as a composite of medical and surgical therapies against cancer.

Long-term aspirin use was defined as patients being prescribed with aspirin for at least 6 months.

Abbreviation: VTE: venous thromboembolism; PE: pulmonary embolism; DVT:deep vein thrombosis; VTE-BLEED: actiVe cancer, male with uncontrolled hyperTension at baseline, anaEmia, history of BLeeding, agE >_60 years, rEnal Dysfunction

| **Table S2 -** **Cancer types in detail after propensity score matching** | | | |
| --- | --- | --- | --- |
|  | **Aspirin**  **(N=423)** | **Non-aspirin**  **(N=846)** | **P value** |
| **Types of cancer - no. (%)** |  |  |  |
| Lung | 63 (14.9) | 131 (15.5) | 0.783 |
| Breast | 25 (5.9) | 48 (5.7) | 0.865 |
| Prostate | 18 (4.3) | 14 (1.7) | 0.005 |
| Uterus | 48 (11.3) | 115 (13.6) | 0.260 |
| Colon | 45 (10.6) | 77 (9.1) | 0.381 |
| Bladder | 20 (4.7) | 29 (3.4) | 0.257 |
| Lymphoma | 19 (4.5) | 38 (4.5) | 1.000 |
| Stomach | 26 (6.1) | 46 (5.4) | 0.607 |
| Ovary | 60 (14.2) | 120 (14.2) | 1.000 |
| Pancreas | 32 (7.6) | 75 (8.9) | 0.432 |
| Brain | 9 (2.1) | 13 (1.5) | 0.447 |
| Others | 58 (13.7) | 140 (16.5) | 0.189 |
